# Perspective: Cellular and Molecular Profiling Technologies in Personalized Oncology

**DOI:** 10.3390/jpm9030044

**Published:** 2019-09-12

**Authors:** Andrea Cruz, Weng Kung Peng

**Affiliations:** International Iberian Nanotechnology Laboratory, 4715-330 Braga, Portugal

**Keywords:** personalized oncology, technological innovations

## Abstract

Cancer is a leading cause of death worldwide and therefore one of the most important public health concerns. In this contribution, we discuss recent key enabling technological innovations (and their challenges), including biomarker-based technologies, that potentially allow for decentralization (e.g., self-monitoring) with the increasing availability of point-of-care technologies in the near future. These technological innovations are moving the field one step closer toward personalized oncology.

Cancer is a leading cause of death worldwide and has been characterized as an heterogeneous disease composed of different cancer cell subtypes, including the mesenchymal cells, endovascular cells, and immune cells [1]. Recent advances in cellular and molecular profiling of common cancers have greatly increased our knowledge on cancer cell evolvability and highlighted the need for early diagnostics and personalized cancer management. One of the paradigms in personalized medicine (PM) is the promise of providing personalized disease diagnosis and management (e.g., prognosis, and predictive treatment/recurrence) as opposed to the traditional ‘one size fits all’ model. The concept of point-of-care and patient-centered healthcare lie at the heart of PM, as medical care is envisioned in the patient’s own ecosystem (‘self-monitoring’) and in decentralized settings (e.g., ‘home diagnostics’) away from hospital (Figure 1).

Traditionally, cancer diagnosis relies on the expensive and low resolution in vivo clinical imaging modalities, such as X-ray, computed tomography, magnetic resonance imaging, ultrasound, and optical imaging, in combination with (often) invasive surgical techniques required for tumor biopsy analysis. In this perspective article, we discuss the emergence of recent key enabling technological innovations (and challenges), including biomarker-based technologies, that potentially allow decentralized diagnostics in the near future. A biomarker is defined as a measurable biological parameter (e.g., DNA, protein) that is indicative of the disease state of the patient and should be highly sensitive, specific, and reproducible [2]. Biomarkers, however, were often found to be lacking in specificity (e.g., in prostate cancer) for disease diagnosis or monitoring (refer to Table 1). The concept of decentralization discussed in this article limits (or rather focuses) the discussion on ´self-monitoring´ (or home diagnostics) rather than facilities offered by companies (e.g., Foundation One). This is one step closer toward personalized oncology.

In recent years, significant advances in the semiconductor industry enabled the emergence of a low cost, portable, micro nuclear magnetic resonance (micro NMR) spectrometer using a single chipset for medical point-of-care diagnostics [3]. These diagnostics include immunomagnetic labeling-based profiling of tumor cells (e.g., breast and colon cancer [4,5], epithelial [6], and melanoma [7]) and the label-free detection of various pathological states, such as oxygenation [8,9]/oxidation [10] level of the blood, malaria screening [11,12], and rapid phenotyping of diabetes mellitus [13,14]. The immunospecific magnetic nanoparticles (MNPs) are typically much smaller (tens of nm) than those of the larger beads used for immunoseparation, and these MNPs are superparamagnetic rather than ferromagnetic. In contrast to ferromagnetic particles, paramagnetic particles do not retain their magnetization outside the influence of an external magnetic field. Positive samples contain MNP-labeled cells, which cause faster dephasing among the spin–spin interaction due to local magnetic fields created by MNPs.

The new paradigm of lowered engineering barriers (e.g., lower cost, higher portability) [3] also opens up a new window of opportunity for in vitro diagnostic targeting on the use of minimally invasive liquid biopsies [15]. A liquid biopsy is the sampling and analysis of nonsolid biological tissue. Liquid biopsy is a promising alternative approach to a direct solid tumor biopsy. It provides minimally invasive and multiple snapshots of the patient as compared to a solid biopsy that only provides one static analysis, which often does not reflect the dynamic of the tumor (e.g., progression) (Table 1).

Furthermore, the existence of tumor heterogeneities and cellular subpopulations have highlighted the strong limitations on the predictive power and diagnostic potential of different imaging modalities, and the issue represents a huge challenge for the development of new diagnostic technologies. As the scientific community learns more about the mechanisms of cancer, new diagnostic tools are being developed to access the vast Omics database, which includes the genomic, transcriptomic, proteomic, and metabolomic landscapes and evolvability of different cancers. However, the possibility of identifying a large number of genetic, protein, or metabolic alterations also has some contraindications, since only some of these alterations will contribute to tumor development. Nevertheless, the improved understanding of the underlying cellular and molecular signatures of cancer has made it possible to develop minimally or noninvasive and cost-effective diagnostic tests potentially applicable in decentralized clinical settings.

The liquid biopsy of periphery blood offers valuable information (e.g., circulating tumor cells (CTCs), circulating DNA (ctDNA), and exosomes), and scientists are already accumulating evidence suggesting its clinical utilities as a novel biomarker in close association with tumor initiation and progression [19]. One of the limitations, however, is the low natural abundance of CTCs in peripheral blood. With an estimated 1–10 CTCs per mL of blood, this is equivalent to the odds of finding less than 10 cells present in 5 million red blood cells [20,21].

Various microfluidic technologies had been developed to ‘sieve’ the CTCs (e.g., enrich, isolate) from the complex of blood matrixes [22]. These microfluidic technologies were designed and developed based on the distinctive physical features of CTCs (e.g., size, density, and deformability) [22,23,24,25] and/or the presence of surface markers (e.g., epithelial cell adhesion molecule, EpCAM) [22,26,27] of the target cells. Studies have reported that the use of microfluidic-based technologies were successful in isolating CTCs from blood samples of patients with metastatic lung, prostate, pancreatic, breast, and colon cancer [22,28,29].

Interestingly, a recent report by Ghazani et al. compared the diagnosis outcome of bulk tumors and CTCs in peripheral blood samples using the micro NMR system, and only a weak correlation was found between each paired sample, suggesting that using CTCs as liquid biopsies and proxies to metastatic solid lesions could be misleading [6]. In a separate study on melanoma tumor samples, Gee et al. found a correlation between melanoma CTCs in peripheral blood and fine needle aspirate, prompting the suggestion of setting up a reference tumor evaluation method using the micro NMR system [7]. Liquid biopsies have also been criticized because the lack of tumor cells in the blood does not necessarily mean that the tumor has not metastasized yet. The efflux of tumor cells into the blood stream is not consistent and happens only occasionally (Table 1).

On one hand, technologies, such as the digital polymerase chain reaction (ddPCR) and next generation sequencing (NGS), used to unravel the genetic profile of cancer cells are quickly emerging, with several advantages in comparison to conventional technologies (as detailed in Table 2 and Table 3). The term digital polymerase chain reaction was first termed by Kinzlerand and Vogelsteinin in mid-1999 to describe a disruptive methodology for the detection of ras oncogene mutations in the stools of patients with colorectal cancer [30]. Using nanoliter-sized sample droplets, ddPCR provides molecular amplification and quantification of low levels of ctDNA and allelic variants in complex body fluids that are usually not detected by conventional real-time PCR [31,32]. Moreover, the analysis of ctDNA by ddPCR, during post-treatment surveillance of head and neck cancer patients, demonstrated that ddPCR can overcome the insufficient accuracy of imaging technologies for early detection of relapsing diseases [32] (Table 2).

Several genomic alterations have been observed within tumors [39,40], requiring a technology able to rapidly probe the entire tumor’s genome. NGS, also known as high-throughput sequencing, can enable the identification of all genetic information within the same tumor sample more quickly and cheaply than classical Sanger sequencing technologies. NGS allows for the identification of high-risk genes associated with hereditary cancers and helps clinicians to diagnose and select patients for effective therapeutic strategies more efficiently [41,42]. The use of NGS for point-of-care oncology has become a reality in some countries (e.g., in Canada) [43] (Table 3). Nevertheless, the implementation of NGS in the clinical setting will also imply the generation of hundreds of gigabytes of data, which need to be analyzed and managed.

The paradigm shift of personalized oncology medicine sees the marriage between novel technologies and the discovery of novel biomarkers. In the near future, we will witness the identification of an increasing number of biomarkers from the genome (e.g., the *p51* gene) to the epigenome (e.g., DNA methylation). This will require the development of machine learning in the form of mathematical models that are capable of making a reliable prediction (e.g., higher accuracy, increased sensitivity and specificity) for disease diagnostics and management (e.g., prognosis, monitoring, and therapeutic efficacy) in a personalized manner. Recently, artificial intelligence in the form of machine learning models (e.g., deep neural networks) have been trained to interpret images of skin and breast cancer in cancer diagnosis [44]. Radiologists are using multidimensional and multiparametric imaging analyses to extract specific features that were previously not possible without machine learning [45]. Multiparametric imaging is a method to obtain three-dimensional images by combining T_2_-weighted, diffusion weighted, and the dynamic contrast-enhanced image.

We foresee that future technologies will feature multiparametric, low-cost, portable spectroscopy-based technologies (e.g., NMR, electron spin resonance, Raman spectroscopy, THz imaging) focusing on the rapid molecular phenotyping of biofluids [14,46], which will be covered in the other articles of this Special Issue [47]. This molecular fingerprint would be time- and patient-specific and would be used (with the help of machine learning) to decipher the disease sub-states with much higher precision. Modern medicine should therefore take advantage of the new generation of technological innovations (especially at the cellular and molecular level) that can deliver advanced diagnostics and monitoring, reduce the clinical burden by using minimally invasive body fluids, and feed results into electronic patient records, subsequently improving the well-being of the patient [48].

## Figures and Tables

**Figure 1:**
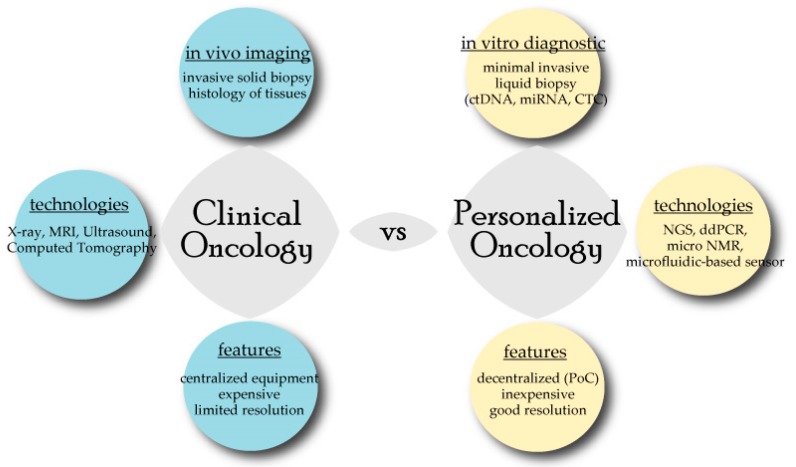
Personalized oncology is correlated with the emergence of key enabling technologies that potentially allow for point-of-care (PoC) testing and decentralization (e.g., self-monitoring), in contrast to traditional clinical diagnostics. The short-forms used were; circulating tumour DNA (ctDNA), microRNA (miRNA), Circulating Tumour Cells (CTC), Next Generation Sequencing (NGS), digital Polymerase Chain Reaction (ddPCR), Nuclear Magnetic Resonance (NMR), Magnetic Resonance Imaging (MRI).

**Table 1:** Comparison between solid biopsy and liquid biopsy [16,17,18].

	Solid Biopsy	Liquid Biopsy
**Overview**	A solid biopsy is taken directly from within a tumor. Generates a picture of the mutations found directly within an individual tumor.	A liquid biopsy analyzes the tumor-related particles that are shed into the bloodstream by all tumors (including by metastasis) present in a patient. This includes the cell-free or complex nucleic acids, such as circulating cell-free DNA (cfDNA) and circulating tumor cells (CTCs). Provides a full picture of all other possible mutations found in all tumors (if the cancer spreading has taken place).
**Advantages/Disadvantages**	Time- and labor-intensive procedure; Localized sample of tissue—some mutations that are found only in a small area of a tumor can be missed or may not provide a complete picture of all the mutations, especially in cases where the cancer has spread beyond the organ of origin; Not easy to be obtained (accessibility); Pain—high; Invasive—high; Pathologic examination of a biopsy can determine whether a lesion is benign or malignant and can help differentiate between different types of cancer.	Quick; Comprehensive tissue profile—all mutations may not be equally represented by cfDNA, which cannot be shed into the bloodstream, or mutations that are found only in a small area of a tumor; Easily obtained; Pain—minimal; Invasiveness—minimal; Low natural abundance—difficult to diagnose; Absence of ctDNA in early stage tumors.

**Table 2:** PCR methodologies at a glance [33,34,35].

	Classical PCR	qPCR	Digital PCR (ddPCR)
**Overview**	Measures the amount of accumulated PCR product at the end of the PCR reaction, at the plateau. Semiquantitative—through comparing the intensity of the amplified band on the gel to standards of a known concentration.	Measures the PCR amplification at the end of each cycle at the exponential phase. Relative quantification—the data are collected during the exponential (log) phase of PCR when the quantity of the PCR product is directly proportional to the amount of template nucleic acid. It is necessary to have DNA from reference genes or standards.	Partitioning a sample into many individual qPCR reactions that run in parallel; some of these reactions contain the target molecule (positive) while others do not (negative). Measures the fraction of negative replicates to determine absolute numbers of copies. Quantitative—the fraction of positive versus negative PCR reactions is used to count the number of target molecules.
**Application examples**	Amplification of DNA for: Sequencing; Genotyping; Cloning.	Quantitative gene expression analysis; Microarray verification; Single nucleotide polymorphisms (SNP) genotyping; Copy number variation; MicroRNA analysis.	Absolute quantification of gene expression; Absolute quantification of next generation sequencing (NGS) libraries; Rare allele detection; Gene copy number.
**Advantages/Disadvantages**	Poor Precision; Low sensitivity; Low resolution; Lower dynamic range (<2 logs); Size-based discriminatory only; Post-PCR processing.	Higher precision; Higher sensitivity; Requires gene references or standard curves; No post-PCR processing; Large dynamic range; Higher throughput, automation compatibility; Highly flexible (users can change reaction volume, throughput, and detection method).	Improved precision and lower errors; Greater discrimination between similar sequences; No need to rely on references or standards—absolute measurements; Desired precision can be achieved by increasing total number of PCR replicates; Capable of analyzing complex mixtures; Allows for small fold change differences to be detected; Greater sensitivity for rare mutation detection; Very low sample volume.

**Table 3:** The development of gene sequencing technologies [36,37,38].

	Sanger Sequencing	Next Generation Sequencing (NGS)
**Overview**	Sanger Sequencing is a sequencing method developed by Frederick Sanger in 1977 to determine the precise nucleotide order of a given DNA fragment. It only sequences a single DNA fragment at a time.	NGS refers to modern high-throughput sequencing processes. It describes a number of different, modern sequencing technologies. NGS is massively parallel, sequencing millions of fragments simultaneously per run.
**Advantages/Disadvantages**	This is a costly process—it takes time, manpower, and more chemicals; Time-consuming—chemical detection and signal detection happens as two separate processes and only one strand can be read at a time; Reliable; This method needs a large amount of template DNA; Generating sequences are lengthier than NGS sequences.	Cheaper process—it reduces time, manpower, and chemicals; High speed—both chemical detection and signal detection of many strands happen in parallel; More accurate; Requires lower amount of DNA; The number of DNA bases per sequenced fragment is lower than the Sanger method; Possibility to detect large number of genetic mutations; however, only some of them will contribute to tumors or the development of disease; Generation of hundreds of gigabytes of data, which need to be analyzed and managed.
**Applications**	Sequencing single gene; Sequencing 1–100 amplicon targets at the lowest cost; Sequencing up to 96 samples at a time without barcoding; Fragment analysis, high-throughput genotyping; Microsatellite or Short Tandem Repeat (STR) analysis; NGS confirmation.	Finding novel variants by expanding the number of targets sequenced in a single run; Sequencing samples that have low input amounts of starting material; Sequencing complete genomes; Detection of variations within an individual genome due to insertions and deletions; Metagenomics studies; Analysis of gene expressions.
